# Evaluation of expert views and considerations to develop rehabilitation sports public services for persons with disabilities in Republic of Korea: a Delphi study

**DOI:** 10.1186/s13690-022-00832-3

**Published:** 2022-03-18

**Authors:** Jiyoung Park, Dongheon Kang, Seon-Deok Eun

**Affiliations:** Department of Healthcare and Public Health Research, Ministry of Health and Welfare, National Rehabilitation Center, National Rehabilitation Research Institute, 58, Samgaksan-ro, Gangbuk-gu, Seoul, Republic of Korea

**Keywords:** Persons with disabilities, Rehabilitation sports, Public service system, Service design method, Design thinking, Republic of Korea, South Korea

## Abstract

**Background:**

Physical activity is required to maintain health; however, resources needed for physical activity (e.g. facilities, instructors, and programmes) are scarce for persons with disabilities (PWD), particularly those who require rehabilitation following hospital discharge and those with severe disabilities. The Republic of Korea presently lacks a legal and administrative framework that supports the implementation of health services for PWD. Therefore, it is necessary to develop such a framework based on the perspectives of PWD, medical and physical education experts, facility managers, and government organisations. Thus, this study aimed to establish expert consensus on the development of rehabilitation sports public services (RSPS) in the Republic of Korea by reviewing previous studies and expert statements.

**Methods:**

Using the Delphi method, we reviewed the literature to identify the critical roles and factors required for planning efficient RSPS programmes, including coordinators, physicians, facility managers, rehabilitation exercise and physical education instructors, and integrated information systems for facilities, instructors, and programmes. We developed a Delphi questionnaire with closed-ended questions, based on the factors derived from the literature review and supplementary open-ended questions, which was administered to a panel of 26 experts.

**Results:**

The panel reached a consensus on most factors (i.e. coordinators, physicians, facility managers, rehabilitation exercise and physical education instructors, and integrated information systems for facilities, instructors, and programmes). However, no consensus was reached regarding ‘when an applicant can continue/discontinue an RSPS programme’, ‘establishing information systems to manage physical fitness of PWD’, and ‘joint operation of the to-be-established system by the Ministry of Health and Welfare and Ministry of Culture, Sports, and Tourism’, leaving room for further debate.

**Conclusions:**

By identifying the factors and roles necessary for RSPS, this study is expected to offer valuable information for state-led pilot projects and contribute to promoting physical activity and quality of life among PWD.

## Background

The personal and social interest in health management and promotion for persons with disabilities (PWD) is increasing. In the Republic of Korea, the proportion of persons with chronic diseases was 84.3% [1], and the obesity rate of PWD was 39.5%, which was higher than the overall obesity rate of 31% for adults in the same year [2, 3]. Physical activity is beneficial for preventing and treating diseases that result from a sedentary lifestyle and assists in the recovery of residual functions for the PWD [4–7]. According to the results of the 2020 Sports-for all Survey [8] for PWD in the Republic of Korea, exercise is ‘effective for health promotion (66.2%)’ and is helpful ‘for physical vitality (71.9%), to have self-esteem (50.8%), and to feel a sense of achievement (54.8%)’. The participation of PWD in sports activities may significantly contribute to the establishment of self-esteem and identity by reducing social, environmental, and personal stress and improving their quality of life [9]. Although the proportion of PWD in the total population is approximately 5%, they account for 16.2% of the total medical expenses [3, 10]. The frequency of hospital visits of PWD in sports activities was lower than that of non-participants, and participation in sports activities was a factor in reducing medical expenses [2]. According to the World Health Organization, PWD are more vulnerable to poor overall health and are at risk of developing secondary disabilities and complications [11]. Additionally, data from the National Health Insurance Service of the Republic of Korea showed that smoking and high body mass index were significantly associated with mortality among PWD, 65.1% of whom were physically inactive [12]. Low physical activity levels among PWD led to a loss of muscle mass and strength and a decreased range of motion (ROM) [13].

Therefore, the Republic of Korea established the 5th Comprehensive Policy Plan for PWD (2018–2022) [14] to realize ‘an inclusive society where PWD can live independently’. The Ministry of Culture, Sports, and Tourism (MCST) proposed establishing a sports-education-welfare linkage system through ‘Sports Vision 2030’ [15]. However, despite the policies and efforts of each ministry, the participation rate of PWD in sports and physical activities is only half of that of persons without disabilities.

PWD who are discharged from care facilities encounter difficulties in performing day-to-day activities, adapting to physical changes, and handling psychological stress. They may continue to grapple with these issues for extended periods and only eventually achieve their desired rehabilitation outcomes. However, there is a lack of adequate support programmes and personnel specialising in healthcare for those with disabilities, as well as resources that promote the physical and psychological adjustment of recently discharged patients with disabilities.

The MCST in the Republic of Korea has launched sports-for-all services—event-oriented sports services—for selected types of sports to promote the health and recreational activities of PWD. However, PWD with a long onset period mainly participate in these services, and the needs of those who are immediately discharged from hospitals are not met by these services. Therefore, physical activity services for PWD that address this issue are needed. Nevertheless, the area currently in charge of that role is not active [16].

Thus, to motivate PWD to enhance their physical, mental, and social abilities, the Republic of Korean government introduced the Act on Guarantee of Right to Health and Access to Health Services for PWD (hereinafter ‘PWD Health Rights Act’) in December 2017. Although the PWD Health Rights Act, led by the Ministry of Health and Welfare (MHW), emphasises rehabilitation sports public services (RSPS), these services have not been implemented due to the absence of a systematic framework and government-level support. Therefore, this study aimed to compile expert opinions and determine the consensus on factors influencing the implementation of practical and systematic RSPS using the Delphi method.

## Methods

**Fig. 1:**
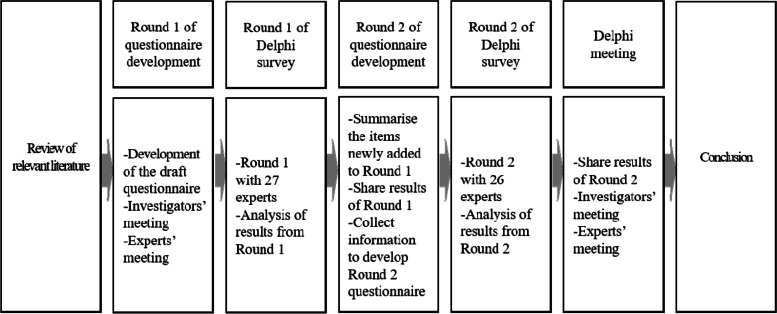
Steps in the Delphi method

The Delphi technique involves a group of experts working together to address complex issues. This method has been used to effectively improve decision-making in the healthcare and social welfare sectors [17, 18]. In the present study, a Delphi survey was conducted in two stages (Fig. 1). In the exploration stage (Round 1), an unstructured questionnaire was used to gather the experts’ opinions. In Round 2, we employed a structured questionnaire as it enables researchers to survey a group simply and systematically [19]. We used a two-stage process because it has been demonstrated as sufficient to achieve the purpose of a Delphi study [20].

### Expert panel selection

We conducted the Delphi survey to collect the opinions of those who provided physical activity programmes to PWD. We used a snowball sampling method to select experts in rehabilitation sports for PWD who had experience providing physical activity programmes to PWD. We approached 31 experts comprising professionals in physical education and special physical education (*n* = 10), rehabilitation medicine doctors (*n* = 11), and other professions, such as professors in social welfare departments, heads of social welfare organizations, personal instructors for PWD, and employees of organizations that provide fitness-related services to PWD (*n* = 10). The response rates in Rounds 1 and 2 were 87% (27 participants) and 96% (26 participants), respectively. Finally, 26 experts participated, and regarding education level, 18 participants (69%) had doctoral degrees, 5 participants had master’s degrees (19%), and 3 participants had bachelor’s degrees (12%). The mean work experience was 20.5 ± 8.03 years (Table 1).

**Table 1:** The composition of experts who participated in the Delphi survey

Classification	Major	Primary disability part	Position	ID	Degree	Major field experience (year)
Academic expert in Kinesiology and Adapted Physical Activity (*n* = 10)	Adapted Physical Activity	Developmental Disability	Professor	E-1	PhD	15
	Adapted Physical Activity	Developmental Disability	Senior researcher	E-2	PhD	17
	Adapted Physical Activity	Developmental Disability	Professor	E-3	PhD	20
	Kinesiology	Physical Disabilities	Professor	E-4	PhD	17
	Adapted Physical Activity	Physical Disabilities	Professor	E-5	PhD	27
	Adapted Physical Activity	Brain lesion Disabilities	Professor	E-6	PhD	30
	Adapted Physical Activity	Physical Disabilities	Professor	E-7	PhD	17
	Adapted Physical Activity	Developmental Disability	Professor	E-8	PhD	22
	Adapted Physical Activity	Physical Disabilities	Senior researcher	E-9	PhD	10
	Adapted Physical Activity	Developmental Disability	Professor	E-10	PhD	27
Academic expert in Rehabilitation Medicine (*n* = 7)	Rehabilitation Medicine	Brain lesion Disabilities	Director	M-1	MS	14
	Rehabilitation Medicine	Brain lesion Disabilities	Professor	M-2	MD	23
	Rehabilitation Medicine	Brain lesion Disabilities	Director	M-3	MD	21
	Rehabilitation Medicine	Brain lesion Disabilities	Director	M-4	MD	30
	Rehabilitation Medicine	Brain lesion Disabilities	Professor	M-5	MS	17
	Rehabilitation Medicine	Physical Disabilities	Professor	M-6	MD	20
	Rehabilitation Medicine	Brain lesion Disabilities	Director	M-7	BS	7
Other related workers (*n* = 9)	Social welfare	Physical Disabilities	Secretary General	O-1	MS	9
	Adapted Physical Activity	Physical Disabilities	Director	O-2	PhD	30
	Adapted Physical Activity	Physical Disabilities	Director	O-3	PhD	25
	Adapted Physical Activity	Developmental Disability	Manager	O-4	MS	11
	Social welfare	Developmental Disability	Professor	O-5	PhD	40
	Social welfare	Physical Disabilities	Director	O-6	PhD	33
	Kinesiology	Physical Disabilities	Manager	O-7	BS	12
	Kinesiology	Physical Disabilities	Manager	O-8	MS	15
	Kinesiology	Physical Disabilities	Manager	O-9	BS	25

### Delphi survey questionnaire

**Fig. 2:**
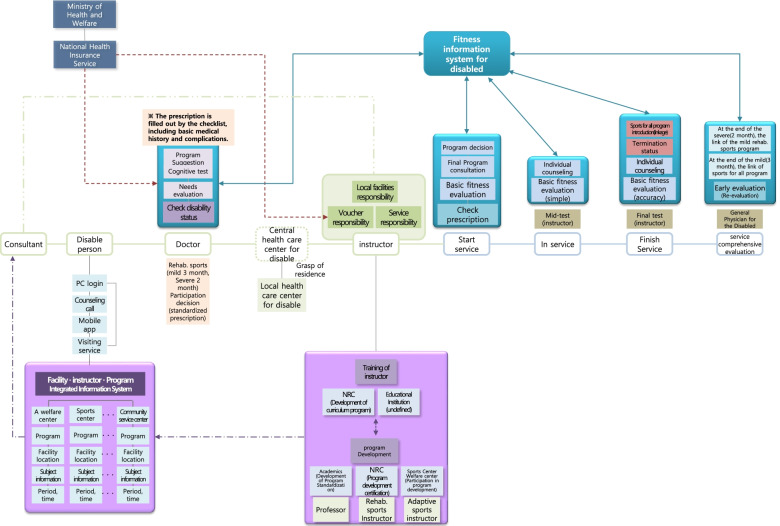
Preliminary RSPS using the Design thinking

**Fig. 3:**
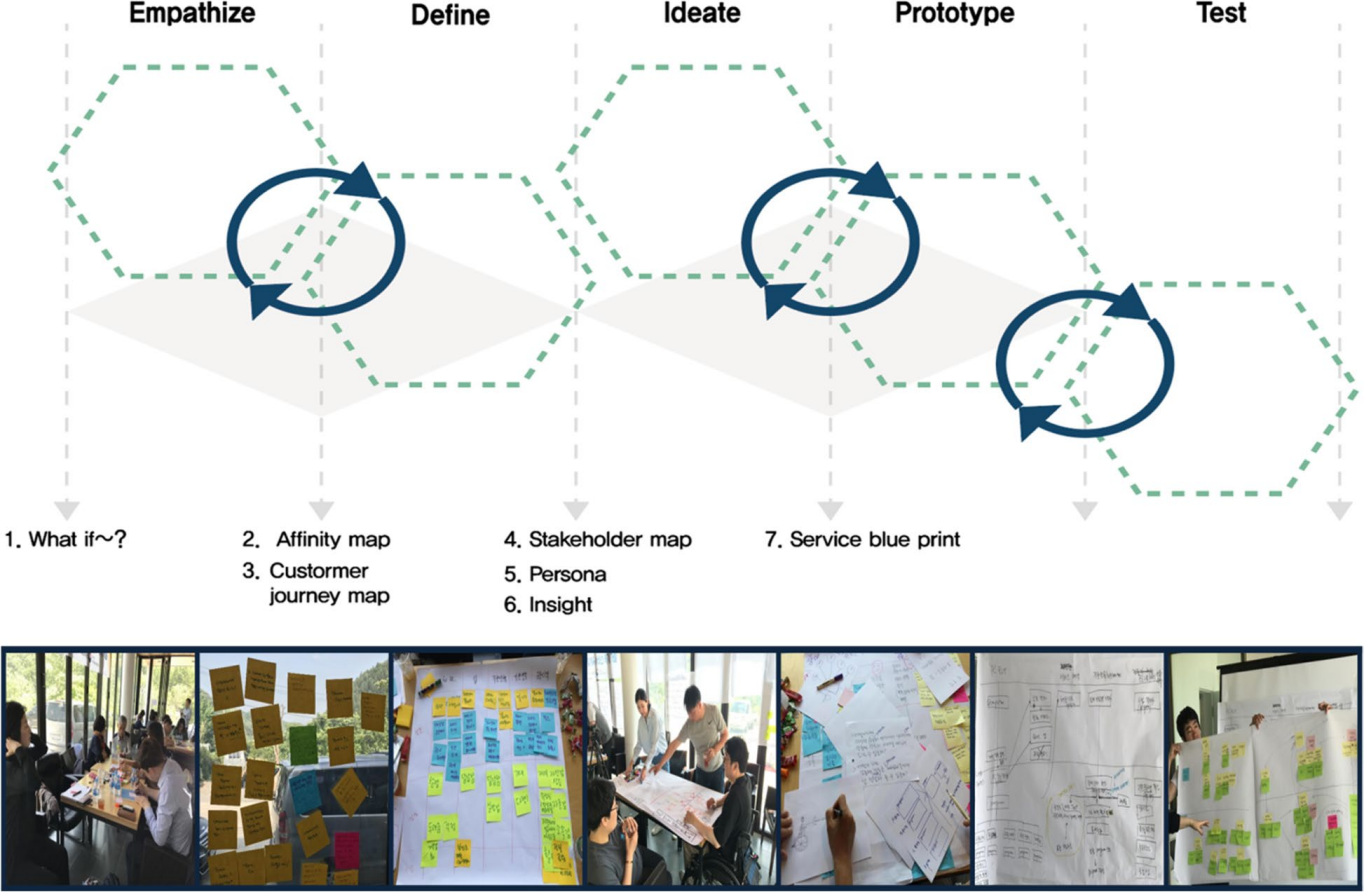
Design thinking process for preliminary RSPS

The questionnaire was designed based on preliminary RSPS (Fig. 2) created through design-thinking methods. PWD, medical and physical education experts, facility managers, and government organizations proposed the preliminary RSPS by the design thinking process (Fig. 3). Design thinking can be effective in providing innovative solutions for policy management and public services [21, 22]. It gives the optimal solution derived from the needs of a small number of people through repeated verification by induction and deduction [23]. Four professors and six researchers in rehabilitation sports who were familiar with the purpose of rehabilitation sports in the Health Rights Act reviewed the Delphi questionnaire that was developed from preliminary RSPS and the literature review results. Each survey statement and domain was presented as a closed-ended question. To overcome the limitations of closed-ended questions, an open-ended question was added to each item, seeking respondents’ free comments. All closed-ended questions used a 5-point Likert scale (1 = strongly disagree, 2 = disagree, 3 = neutral, 4 = agree, and 5 = strongly agree). The Delphi questionnaire was distributed to the panel via email in each round. In Round 1, the questionnaire comprised questions representing the following eight domains: 1) roles and duties of a coordinator, 2) roles and duties of a physician, 3) roles and duties of a sports facility manager, 4) roles and duties of a rehabilitation sports instructor, 5) integrated system on facilities, instructors, and programmes (tentative name), 6) information system on the physical fitness of PWD (tentative name), 7) access to RSPS for PWD, and 8) implementation of RSPS. The participants were asked to rate the necessity of each item, representing a factor influencing the establishment of a framework for RSPS. Moreover, they were requested to provide additional comments on each item (Table 2).

**Table 2:** Delphi questionnaire structure

Essential elements of the questionnaire	Question type & Number of items		
	Round 1		Round 2
	Closed-ended	Open-ended	Closed-ended
Roles and duties of a coordinator	8	3	12
Roles and duties of a physician	22	3	28
Roles and duties of a sports facility manager	4	1	4
Roles and duties of a rehabilitation sports instructor	19	3	21
Integrated system on facilities, instructors, and programmes (tentative name)	8	2	14
Information system on the physical fitness of PWD (tentative name)	9	3	11
Access to RSPS for PWD	2	–	2
Implementation of RSPS	–	3	12

### Statistical analysis

Open-text comments were analysed using the NVivo programme (QSR International), which is used for qualitative data analysis. Three experts with doctoral degrees reviewed words occurring more than twice to ascertain whether the inclusion of additional factors would be necessary for Round 2. Analysis of responses to closed-ended questions was performed upon completion of each round using SPSS 21. The results of the descriptive statistics were measured as mean and standard deviation, and central tendency statistics as median and mode. As a measure of content validity index (CVI) and a relative measure of statistical dispersion, a positive coefficient of variation (CV) was calculated as the standard deviation divided by the mean (see Table 3) [19]. The stop criterion includes a CVI ≥ .75 (75%) based on a valid percentage of responses with a mean score ≥ 4.0 [18, 24] and positive rates such as the CV. This is the value at which it can be judged that the opinions of several participants in the survey are agreed upon [18, 24]. We used mean ≥ 4.0, CVI ≥ .75 (75%), and CV < .50 [18, 24] as the stop criteria. Panel members were requested to provide their feedback to statements that met these thresholds, and further adjustments were made with the panel’s consent, where necessary, in Round 2.

**Table 3:** Major terms used in the Delphi method

Term	Definition
Mean	The sum of values in the panel members’ responses to each item is divided by the number of members. Stop criterion: mean value ≥4.0.
Standard deviation	As a measure of dispersion, standard deviation indicates the degree of spread in the panel members’ responses from the mean value and is calculated as the square root of the deviation of each item.
CVI	A valid percent of the panel members’ responses with a score of 4 or more (positive rate). Stop criterion CVI value ≥ .75
CV	As a measure of dispersion, CV refers to the degree of relative dispersion of the panel members’ responses and is calculated as the standard deviation divided by the mean. Stop criterion CV < .50

## Results

The two survey rounds using the Delphi method revealed that opinions of additional personnel who explained RSPS to the PWD to provide smooth services were needed. Regarding the roles and duties of a coordinator, the statement, ‘identify the physical condition of service applicants and provide them with appropriate information on accessible on facilities, instructors, and programmes’ was found to be appropriate (mean = 4.29, CV = 0.16) and necessary (mean = 3.96, CV = 0.28). However, ‘coordinator’ was not considered an appropriate job title for the position described. The statement exploring where coordinators can be deployed in the context of community-based public organizations had a mean score of < 4, requiring further consideration among the parties involved.

Furthermore, physicians must identify the disability status (mean = 4.88, CV = 0.06) and perform cognitive tests (mean = 4.12, CV = 0.20) to ensure that applicants can perform RSPS. Regarding the statement, ‘making decisions on whether an applicant can continue/discontinue RSPS programmes’, the rehabilitation medicine group perceived physicians as decision-makers. However, the physical education-related group advocated for mutual consent between physicians and instructors to make such a decision. The rehabilitation sports instructor was expected to identify the following through a doctor’s prescription: the seriousness of the disability (type), comorbid diseases and necessary precautions, pain areas associated with disability conditions, functional capacity (e.g. the ability to walk and stand without aid), gait test results, joint ROM, stiffness test results, muscle strength (upper and lower extremities), bone density test results, sensory test results, infectious disease test results (e.g. hepatitis and tuberculosis), and the appropriate type of exercise when participating in rehabilitation sports programmes. Furthermore, they were required to assess the present status of adult diseases (e.g. hypertension and hyperlipidaemia), psychological tests (depression, self-esteem, and quality of life), and the applicant’s intention (willingness and enthusiasm) to engage in sports activities.

The sports facility manager must ensure that applicants present a doctor’s prescription (or opinion), identify when the programmes are offered in the facility, and provide information on costs and how to use the programme. The manager should also submit a list of applicants for the RSPS to the community centre.

The rehabilitation sports instructor must verify the doctor’s prescription (or opinion), perform a motor function test (baseline), and meet with the applicants to select a suitable programme (mean = 4.77, CV = 0.10). Additionally, the instructor must meet with each applicant to determine their needs during the service period (mean = 4.73, CV = 0.09), perform a motor function test at the end of the RSPS programme (post-service), and meet to discuss the completed programme and plans (i.e. whether the applicant must continue or discontinue RSPS) (mean = 4.81, CV = 0.06). Finally, the instructor should submit their opinion on whether the applicant can continue or discontinue RSPS to the physician. At the end of an RSPS programme, the instructor must introduce a new programme related to sports for all projects held by the MCST to help the applicant continue their physical activities. The instructor is also required to report changes in the applicants’ physical condition during RSPS and post-service positive changes regarding their physical, psychological, and social aspects to a prescribed hospital. Although Round 1 survey results revealed the need for a physical performance test during the service period (interim), a consensus was reached with a mean score of 3.5. Among the basic physical qualities of applicants that the instructor evaluates pre-service, ‘body composition’, ‘physical strength’, ‘muscle strength’, ‘flexibility’, ‘muscle endurance’, ‘cardiopulmonary endurance’, and ‘balance’ showed mean scores ≥4. Additionally, the instructor must be familiar with ‘how to assess each type of disability’. The rehabilitation medicine group presented a contrasting opinion, as their mean scores of ‘body composition’, ‘cardiopulmonary endurance’, ‘balance’, and ‘how to assess each type of disability’ were < 4. The factors evaluated in the motor function test were ‘hand function’, ‘gait and lower limb function’, ‘upper limb function’, ‘balance ability’, and ‘ROM’. Of these, ROM was found to be appropriate with mean scores ≥4. However, the rehabilitation medicine group showed mean scores < 4 for these factors, yielding conflicting results with the physical education-related group.

An information system on facilities, instructors, and programmes should be designed to contain the following information: facility location, availability of facilities used exclusively by PWD, programme schedules available in each facility, and availability of rehabilitation sports instructors (differentiated according to the type of disability) being offered by each facility showed mean scores > 4. It should also include a database of graduates of professional rehabilitation sports instructor training courses and rehabilitation sports programmes available for each type of disability (mean scores > 4). Moreover, the system can include information on programmes differentiated by gender and age group, programme fee, the number of applicants on the waiting list and estimated waiting period, applicant ineligibility conditions, and instructions for a new sport (e.g. sports for all) to continue physical activities (mean scores > 4).

The information system on the physical fitness of PWD (tentative name) had a mean score of 4.15, confirming its necessity. This further suggests that the physician must decide whether each applicant can continue or discontinue an RSPS programme. Additionally, all information compiled by the instructor through consultations and tests or opinions about each applicant must be disclosed to the physician. However, the rehabilitation medicine group did not reach a consensus on the necessity of the information system on the physical fitness of PWD (tentative name) (mean score = 3.29). Thus, their perspective differed from that of the physical education-related group. The first survey indicated the need for follow-up monitoring (on every quarter basis for a maximum of 2 years) of applicants who have completed RSPS programmes; however, its necessity was not supported, given the mean score of 3.77. During the questionnaire development process, the concept of selecting the ‘Central Health and Medical Centre for PWD’ as a unit overseeing the two aforementioned information systems (i.e. the integration of facilities, instructors, and programmes; and the physical fitness of PWD) was discussed. However, a consensus was not reached (mean scores of 3.70 and 3.77 in Rounds 1 and 2, respectively), meriting further consideration.

The ideal number of PWD attending RSPS programmes was ≤5 (46.2%). However, the panel suggested the need for not only adaptive programmes that can be customized according to the type of disability and functional capacity of the applicant but also flexible change in the maximum number of trainees, depending on the nature of the programme. Additionally, providing enough information, including expected outcomes, was considered important (mean score = 4.23) to encourage trainees to participate in new sports activities (sports for all) organized by the MCST upon completion of RSPS. Moreover, ‘information sharing between the MHW and the MCST’, ‘exact definitions of rehabilitation sports and sports for all’, and ‘supply of proper assistive devices and equipment’ were considered necessary.

The experts’ responses to Round 1 open-ended questions revealed that, to promote active participation in RSPS programmes among recently discharged patients with disabilities, the following were identified as necessary: ‘enthusiastic invitation from a physician and nurse’, ‘providing promotional materials at community-based organizations such as welfare centres, clinic centres, service centres, and district offices’, ‘distribution of promotional leaflets at the time of discharge’, ‘instructions via cell phones’, and ‘advertising through media platforms’ (Tables 4, 5, 6, 7, 8, 9, 10, 11).

**Table 4:** Survey results for the ‘roles and duties of a coordinator’ domain

Items representing ‘roles and duties of a coordinator’		Round 1 survey results (mean)	Round 2 survey results					
			Overall				Physical education-related group	Rehabilitation medicine group
			mean	SD	CVI	CV	mean	mean
Necessity of a coordinator	3–1-1. Necessity of coordinator’s roles	4.00	3.96	0.73	0.28	5	4.21	3.29
Necessity of existing positions that can act as a coordinator	3–2-1. Rehabilitation sports instructors working at fitness training facilities, including social welfare centres	–	3.23	0.42	0.28	3	3.53	2.43
	3–2-2. People responsible for community-based rehabilitation (CBR) at community clinic centres	–	3.65	0.65	0.28	4	3.53	4.00
	3–2-3. People responsible for social welfare services at community service centres	–	2.96	0.35	0.49	3	2.84	3.29
Necessity of coordinator’s duties and job title	3–3-1. Primary task: Identify the physical condition of the service applicant and provide them with appropriate services about the information system on available facilities, instructors, and programmes	4.17	4.29	0.77	0.16	5	4.42	3.80
	3–3-2. Job title: ‘Coordinator’ is an appropriate job title for the position described in the paragraph of 1–3-1	3.13	3.08	0.35	0.38	2	3.21	2.60
Necessity of a new job title in place of ‘coordinator’	3–4. Necessity of a new job title reflecting duties of a coordinator	–	3.63	0.69	0.26	4	3.89	2.60
Workplace for the coordinator position	3–5-1. Community clinic centre	3.79	3.88	0.73	0.26	4	3.84	4.00
	3–5-2. Community service centre	3.42	3.25	0.42	0.44	5	3.53	2.20
	3–5-3. Welfare centre	3.42	3.13	0.42	0.36	4	3.47	1.80
	3–5-4. Regional associations of PWD	2.42	2.13	0.08	0.42	2	2.16	2.00
Whether it is appropriate to assign the duties of a coordinator to the people responsible for CBR at community clinic centres	3–6. Assign duties of coordinator to CBR personnel	–	3.50	0.54	0.38	5	3.17	4.50

**Table 5:** Survey results for the ‘roles and duties of a physician’ domain

Items representing ‘roles and duties of a physician’		Round 1 survey results (mean)	Round 2 survey results					
			Overall				Physical education-related group	Rehabilitation medicine group
			mean	SD	CVI	CV	mean	mean
Necessity of duties of a physician	4–1-1. Identify disability status	4.74	4.88	0.32	1.00	0.07	4.89	4.86
	4–1-2. Assess the enthusiasm to perform exercises	3.63	3.73	0.92	0.54	0.25	3.79	3.57
	4–1-3. Cognitive test	4.00	4.12	0.85	0.73	0.21	4.05	4.29
	4–1-4. Balance ability test	3.70	3.88	0.99	0.65	0.26	3.74	4.29
	4–1-5. Deciding whether applicants can continue/discontinue an RSPS programme	3.70	3.81	0.95	0.77	0.25	3.68	4.14
Deciding whether applicants can continue/discontinue an RSPS programme	4–2-1. Physician decides	3.22	3.31	1.07	0.50	0.32	3.16	1.07
	4–2-2. Instructor decides	2.85	2.85	0.94	0.15	0.33	3.11	0.94
	4–2-3. Decision is made upon consent from physician and instructor	3.93	4.27	1.02	0.81	0.24	4.58	1.02
Factors to be identified by the instructor in a doctor’s prescription presented by an applicant	4–3-1. Seriousness of disability (type)	4.85	4.88	0.32	1.00	0.07	4.89	0.32
	4–3-2. Comorbid diseases and precautions to be taken	4.81	4.85	0.32	1.00	0.07	4.89	0.32
	4–3-3. Pain areas associated with disability conditions	4.67	4.77	0.37	1.00	0.08	4.84	0.37
	4–3-4. Functional capacity (e.g. the ability to walk and stand without aid)	4.41	4.54	0.70	0.92	0.15	4.47	0.70
	4–3-5. Gait test results	4.15	4.35	0.75	0.85	0.17	4.32	0.75
	4–3-6. Joint range of motion	4.33	4.38	0.82	0.81	0.19	4.32	0.82
	4–3-7. Stiffness test results	4.26	4.31	0.79	0.81	0.18	4.21	0.79
	4–3-8. Muscle strength (upper and lower extremities)	4.15	4.23	0.94	0.77	0.22	4.11	0.94
	4–3-9. Bone density test result	3.85	4.00	0.97	0.65	0.24	4.05	0.97
	4–3-10. Electrocardiographic stress test	3.89	3.92	0.91	0.65	0.23	3.95	0.91
	4–3-11. Sensory test results	3.96	4.04	0.82	0.69	0.20	4.00	0.82
	4–3-12. Cognitive test results	3.93	3.92	0.85	0.62	0.22	3.95	0.85
	4–3-13. Infectious disease test (e.g. hepatitis, tuberculosis)	4.30	4.42	0.77	0.85	0.18	4.53	0.77
	4–3-14. Recommend the type of exercise appropriate for applicants participating in rehabilitation sports programme	3.96	4.23	0.98	0.77	0.23	4.21	0.98
Additional factors that the instructor should identify from a doctor’s prescription	4–4-1. The present status of adult diseases (e.g. hypertension and hyperlipidaemia)	–	4.50	0.61	0.89	0.14	4.58	0.61
	4–4-2. Psychological test (depression, self-esteem, and quality of life)	–	4.19	0.67	0.81	0.16	4.32	0.67
	4–4-3. Origin of disability	–	3.65	0.90	0.46	0.25	3.58	0.90
	4–4-4. Disability prognosis (expected progression)	–	3.77	0.91	0.62	0.24	3.95	0.91
	4–4-5. Applicants’ intention (willingness and enthusiasm) to participate in sports activities	–	4.04	0.83	0.81	0.21	4.16	0.83
	4–4-6. The presence/absence of family support	–	3.77	0.91	0.62	0.24	3.95	0.91

**Table 6:** Survey results for the ‘roles and duties of a sports facility manager’ domain

Items representing ‘roles and duties of a sports facility manager’		Round 1 survey results (mean)	Round 2 survey results					
			Overall				Physical education-related group	Rehabilitation medicine group
			mean	SD	CVI	CV	mean	mean
Task appropriateness of sports facility manager	Ensures that applicants present a doctor’s prescription (or opinion)	4.62	4.69	0.76	0.96	0.16	4.63	4.86
	5–1-2. Identifies when the programmes are offered within the facility	4.69	4.73	0.58	0.96	0.12	4.68	4.86
	5–1-3. Provides information on cost of services	4.46	4.54	0.69	0.92	0.15	4.42	4.86
	5–1-4. Submits the list of applicants for RSPS to a community centre	4.38	4.46	0.67	0.92	0.15	4.32	4.86

**Table 7:** Survey results for the ‘roles and duties of a rehabilitation sports instructor’ domain

Items representing roles and duties of a ‘rehabilitation sports instructor’		Round 1 survey results (mean)	Round 2 survey results					
			Overall				Physical education-related group	Rehabilitation medicine group
			mean	SD	CVI	CV	mean	mean
Task appropriateness of rehabilitation sports instructor	6–1-1 Checks the doctor’s prescription (or opinion), performs a quick motor function test, and meets with the applicant to choose a programme for them	4.44	4.77	0.50	0.96	0.11	4.84	4.57
	6–1-2. Meets with each applicant to determine their needs during the service period	4.48	4.73	0.42	1.00	0.09	4.79	4.57
	6–1-3. Performs a motor function test at the end of RSPS (post-service) and holds a meeting to discuss the programme completed and future plans (i.e. whether the applicant can continue/discontinue RSPS)	4.48	4.81	0.32	1.00	0.07	4.89	4.57
	6–1-4. At the end of RSPS, introduces a new programme effective as sports for all	4.67	4.85	0.23	1.00	0.05	4.95	4.57
	6–1-5. Submits opinion on whether the applicant can continue/discontinue RSPS to the physician	4.33	4.69	0.48	1.00	0.10	4.68	4.71
Additional factors on task appropriateness of rehabilitation sports instructor	6–2-1. Reports to a prescribed hospital changes in the applicant’s physical condition that have occurred during RSPS	–	4.42	0.69	0.89	0.16	4.42	4.43
	6–2-2. Can identify and assess post-service positive changes in physical, psychological, and social aspects	–	4.00	0.66	0.77	0.16	4.11	3.71
The need for a physical performance test during the service period (interim)	6–3. Conducts a physical performance test during the service period (interim)	–	3.50	1.21	0.50	0.35	3.74	2.86
Appropriateness of basic physical qualities assessed	6–4-1. Body composition	4.15	4.42	0.60	0.89	0.14	4.63	3.86
	6–4-2. Muscle strength	4.56	4.65	0.54	0.89	0.12	4.79	4.29
	6–4-3. Flexibility	4.15	4.35	0.90	0.81	0.21	4.47	4.00
	6–4-4. Muscle endurance	4.22	4.35	1.12	0.85	0.26	4.47	4.00
	6–4-5. Cardiopulmonary endurance	4.11	4.23	0.84	0.81	0.20	4.42	3.71
	6–4-6. Speed	3.22	3.19	1.18	0.31	0.37	3.21	3.14
	6–4-7. Balance	4.04	4.08	0.96	0.73	0.24	4.16	3.86
	6–4-8. Power	3.37	3.27	1.18	0.35	0.36	3.21	3.43
Additional factors on the appropriateness of basic physical qualities assessed	6–5-1. Assessment method for the type of disability	–	4.04	4.04	0.73	0.15	0.151	2.71
Appropriateness of motor function test items	6–6-1. Hand function	3.96	4.04	0.82	0.69	0.20	4.32	3.29
	6–6-2. Gait and lower limb function	4.26	4.31	0.77	0.77	0.18	4.58	3.57
	6–6-3. Upper limb function	4.19	4.35	0.60	0.85	0.14	4.63	3.57
	6–6-4. Balance ability	4.15	4.12	0.76	0.77	0.19	4.37	3.43
	6–6-5. ROM (Range of Motion)	4.00	4.19	0.69	0.81	0.17	4.42	3.57

**Table 8:** Survey results for ‘an integrated information system on facilities, instructors, and programmes (tentative name)’ domain

Items related to an integrated information system on facilities, instructors, and programmes (tentative name)		Round 1 survey results (mean)	Round 2 survey results					
			Overall				Physical education-related group	Rehabilitation medicine group
			mean	SD	CVI	CV	mean	mean
Appropriateness and necessity of an integrated information system on facilities, instructors, and programmes (tentative name)	7–1-1. Necessity of establishing an integrated information system on facilities, instructors, and programmes (tentative name)	4.56	4.69	0.37	0.96	0.08	4.84	4.29
	7–1-2. Facility location information	4.67	4.81	0.37	1.00	0.08	4.84	4.71
	7–1-3. Availability of facilities used exclusively for PWD	4.33	4.38	0.76	0.85	0.17	4.37	4.43
	7–1-4. Programme schedules	4.78	4.77	0.45	1.00	0.10	4.74	4.86
	7–1-5. Information on availability of rehabilitation sports instructors	4.70	4.85	0.32	1.00	0.07	4.89	4.71
	7–1-6. Information on programmes (differentiated according to type of disability) being offered by each facility	4.89	4.92	0.23	1.00	0.05	4.95	4.86
	7–1-7. Information on the graduates of professional instructor training courses	3.93	4.12	0.91	0.81	0.22	4.05	4.29
	7–1-8. Information on rehabilitation sports programmes available for each type of disability	4.52	4.65	0.96	0.96	0.21	4.63	4.71
Additional factors on the appropriateness of an integrated information system on facilities, instructors, and programmes (tentative name)	7–2-1. Programmes differentiated by gender	–	4.00	1.05	0.69	0.26	4.00	4.00
	7–2-2. Programmes differentiated by age group	–	4.19	0.56	0.89	0.13	4.26	4.00
	7–2-3. Fee information	–	4.38.	0.76	0.89	0.17	4.37	4.43
	7–2-4. The number of applicants on the waiting list and the estimated waiting period	–	4.19	0.83	0.85	0.20	4.16	4.29
	7–2-5. Applicant ineligibility conditions	–	4.50	0.61	0.92	0.14	4.58	4.29
	7–2-6. Instructions for a new sport (e.g. sports for all) to continue physical activities	–	4.42	0.50	0.92	0.11	4.63	3.86

**Table 9:** Survey results for ‘an information system on the physical fitness of PWD’ (tentative name) domain

Items related to ‘an information system on the physical fitness of PWD’ (tentative name)		Round 1 survey results (mean)	Round 2 survey results					
			Overall				Physical education-related group	Rehabilitation medicine group
			mean	SD	CVI	CV	mean	mean
Necessity of the information system on the physical fitness (capacity) of PWD (tentative name) and appropriateness of associated statements	8–1-1. Establishment of the above system	3.93	4.15	0.70	0.77	0.17	4.47	3.29
	8–1-2. Results of tests conducted by the physician (sharing the applicant’s medical information)	3.59	3.73	0.93	0.69	0.25	4.26	2.29
	8–1-3. Physician’s decision on whether the applicant can continue/discontinue RSPS	4.07	4.23	0.61	0.85	0.15	4.47	3.57
	8–1-4. Disclose the information the physician has compiled on the applicant to the instructor	3.78	3.85	1.07	0.65	0.28	4.16	3.00
	8–1-5. Results of physical performance tests conducted by the instructor	4.37	4.50	0.58	0.89	0.13	4.68	4.00
	8–1-6. Content of consultations conducted by the instructor	4.15	4.23	0.83	0.73	0.20	4.37	3.86
	8–1-7. Instructor’s opinion on whether the applicant can continue/discontinue RSPS	4.15	4.15	0.61	0.85	0.15	4.47	3.29
	8–1-8. Disclose all the information compiled by the instructor to the physician	4.07	4.31	0.77	0.89	0.18	4.47	3.86
Additional factors on the appropriateness of an information system on the physical fitness of PWD (tentative name)	8–2-1. Follow-up monitoring on applicants who completed RSPS (quarterly basis for up to two years)	–	3.77	0.78	0.69	0.21	3.95	3.29
Appropriateness of the Central Health and Medical Centre for Persons with Disabilities as a unit overseeing both information systems (tentatively named) on the integration of facilities, instructors, and programmes and physical fitness of disabled people	8–3. Central Health and Medical Centre for Persons with Disabilities as a system management unit	3.70	3.77	0.96	0.61	0.25	3.84	3.57
Management unit of the information system on the physical fitness of PWD (tentative name)	8–4. Joint operation/management by the medical and physical educational organisations	–	3.81	0.69	0.69	0.18	4.16	2.86

**Table 10:** Survey results for the ‘Access to RSPS for PWD’ domain

Items representing ‘Access to RSPS for PWD’						
Ideal number of trainees for training	Analysis of Round 1 survey results (overall)			Analysis of Round 2 survey results (overall)		
	No.	Agreed (people)	Percentage (%)	No.	Agreed (people)	Percentage (%)
	① PWD ≤ 5	11	40.74	① PWD ≤ 5	12	46.2
	② PWD ≤ 10	5	18.52	② PWD ≤ 10	5	19.2
	③ PWD ≤ 15	1	3.70	③ PWD ≤ 15	0	0
	④ Others	10	37.04	④ Others	9	34.6
	Total	27	100	Total	26	100
Ratio between instructors and trainees for training	Analysis of Round 1 survey results (overall)			Analysis of Round 2 survey results (overall)		
	No.	Agreed (people)	Percentage (%)	No.	Agreed (people)	Percentage (%)
	① 3 PWD: 1 instructor	7	25.93	① 3 PWD: 1 instructor	8	30.8
	② 4 PWD: 1 instructor	1	3.70	② 4 PWD: 1 instructor	3	11.5
	③ 5 PWD: 1 instructor	6	22.22	③ 5 PWD: 1 instructor	4	15.4
	④ Others	13	48.15	④ Others	11	42.3
	Total	27	100	Total	26	100

**Table 11:** Survey results for the ‘implementation of RSPS’ domain

Items representing the ‘implementation of RSPS’		Round 1 survey results (mean)	Round 2 survey results					
			Overall				Physical education-related group	Rehabilitation medicine group
			mean	SD	CVI	CV	mean	mean
Ways to help trainees participate in sports for all after RSPS	10–1-1. Join a peer community	–	3.77	0.98	0.62	0.26	3.79	3.71
	10–1-2. Feedback channel to gather opinions of the instructor	–	3.62	1.16	0.54	0.32	3.68	3.43
	10–1-3. Implement regular monitoring after RSPS	–	3.62	1.24	0.62	0.34	3.74	3.29
	10–1-4. Information sharing between the MHW and the MCST	–	4.00	1.01	0.73	0.25	4.16	3.57
	10–1-5. Develop exact definitions of rehabilitation sports and sports for all	–	4.04	1.05	0.73	0.26	4.11	3.86
	10–1-6. Provide patient and caretaker with sufficient information	–	4.23	0.98	0.85	0.23	4.21	4.29
	10–1-7. Provide appropriate assistive devices and equipment	–	4.00	1.13	0.73	0.28	4.05	3.86
Ways to promote sports for all services	10–2-1. Active invitation from physicians and nurses	–	4.58	0.45	0.92	0.10	4.74	4.14
	10–2-2. Provide promotional materials at community-based organisations such as welfare centre, clinic centre, service centre, and district office	–	4.50	0.69	0.89	0.15	4.58	4.29
	10–2-3. Distribute promotional leaflets at the time of discharge	–	4.62	0.54	0.92	0.12	4.79	4.14
	10–2-4. Provide instructions via mobile phones	–	4.12	0.83	0.73	0.20	4.16	4.00
	10–2-5. Advertise through media platforms	–	4.27	0.63	0.89	0.15	4.21	4.43

## Discussion

In this study, we used a Delphi survey to gather the opinions of some experts on a preliminary framework for RSPS. During the development of the framework based on a literature review and the experiences of PWD and stakeholders, expert consensus was reached on the use of doctors’ prescriptions (or opinions) instead of basic physical performance tests suggested before, during, and after an RSPS programme. As an exception, if the applicant has a severe disability or it is difficult to identify their motor function based on the doctor’s prescription, a motor function test must be performed pre-service. We identified a need to establish an integrated information system that PWD can access via websites, apps, telephone, or consultations with coordinators to obtain information on RSPS in their districts.

Physical activities performed during leisure time in parks and gyms are known to reduce musculoskeletal and neuropathic pain in people with spinal cord injuries, enhance their physical and mental health, and alleviate or prevent related complications [25]. Researchers also report that regular physical activity helps prevent and manage various physical and mental health problems [25, 26]. Sallis [27] describes exercise as a vaccine that is essential for preventing chronic diseases and premature death. Therefore, recently discharged patients with disabilities must present a doctor’s prescription to help them safely and promptly engage in physical activities. The medical and physical education groups had conflicting opinions regarding who should decide whether an applicant can continue or discontinue an RSPS programme. This finding suggests the need for further exploration of real-world practices through a pilot study and subsequent discussion regarding the required adjustments.

The instructor is expected to identify test results and other medical information through a doctor’s prescription. Mood disorders, such as depression and anxiety, resulting from a stroke can be relieved with low-intensity workouts such as yoga [28]. Moreover, physical activity has antidepressant effects that help overcome depressive symptoms [29]. Similarly, this study suggests psychological factors, such as depression, self-esteem, and perceived quality of life, as important in influencing sports activities such as RSPS. Additionally, the applicant’s own intention (willingness) to participate in sports activities has a greater impact on motor skill improvement than being invited by family or friends. Thus, the applicant’s willingness should be assessed before the RSPS programme. Therefore, the physician must provide all the relevant information for the instructor in the prescription.

The rehabilitation sports instructor must be proactive to prevent health and accident risks by focusing on any physical signs that a trainee may develop during RSPS and reporting them to a hospital. This requires the ability to identify and assess physical and psychological changes among RSPS trainees.

The results also suggest the importance of establishing an integrated information system that provides information on accessible facilities, available instructors, and programmes to allow PWD to access RSPS easily. Specifically, there is a need for information on the availability of qualified instructors who can help recently discharged patients with disabilities and provide timely updates on new programmes developed by academics, research and development institutes, and community clinic centres.

Establishing an information system regarding the physical fitness of PWD is necessary to provide easy access to test results that assess pre- and post-RSPS changes in the trainees’ physical or motor functions. Since physical abilities constitute sensitive personal information, physicians, instructors, and facility managers must have distinct levels of authority to access and modify information. Additionally, given that personal information is included, some panel members opposed the establishment of information systems. However, information systems are necessary to provide RSPS systematically and efficiently to promote health among PWD. However, this study does not stipulate how to build an information system. To provide RSPS efficiently in the future, further information system development research is required considering the results of this study. Therefore, the MHW and the MCST must enable PWD to continue their sports activities by participating in sports-for-all services provided by the latter after RSPS.

## Conclusion

This study aimed to develop a framework for the implementation of public services designed to promote exercise and sports activities for the rehabilitation of PWD, consistent with the growing demand for rehabilitation exercise and sports following the Health Rights Act of 2017. A Delphi method was used to gather the opinions of relevant stakeholders, including PWD, service providers, and experts in the medical and physical education sectors. A consensus was reached on most statements representing RSPS; however, panel members disagreed on who should decide on how long an applicant can participate in an RSPS programme. The statements that the panel agreed on must be explored further in terms of the experiences of service recipients and providers by conducting a pilot study on rehabilitation sports. Additionally, further review and discussion are necessary concerning new policies, budgets, and cooperation with other government agencies for the effective implementation of RSPS programmes. Future studies should address in-service education designed to promote the effective roles of the involved parties, including coordinators, physicians, and instructors.

This study may contribute to developing RSPS to effectively bridge the gap between rehabilitation therapy provided in clinical settings after discharge and sports-for-all services led by the MCST and, more importantly, to help PWD to promptly resume day-to-day activities. Continuous endeavours to improve RSPS programmes are expected to promote the health and quality of life of PWD who face difficulties in managing their health. Finally, rehabilitation exercises and sports services for people with newly acquired or severe disabilities are effective in increasing their ability to manage their health and reducing socioeconomic costs by preventing chronic diseases and complications associated with disability.

## Data Availability

The datasets analysed during the current study are available from the corresponding author on reasonable request.

## Abbreviations

CVI: Content validity index
CV: Coefficient of variation
MCST: Ministry of Culture, Sports, and Tourism
MHW: Ministry of Health and Welfare
PWD: Persons with disabilities
ROM: Range of motion
RSPS: Rehabilitation sports public services
